# Single-Nucleus RNA Sequencing Reveals Muscle Fiber Cell Heterogeneity during Human Skeletal Muscle Aging

**DOI:** 10.1093/geroni/igaf122.235

**Published:** 2025-12-31

**Authors:** Li Wu, Caixia Gong, Ange Wang, Jing Chen, Qin Zhang

**Affiliations:** Zhejiang University School of Medicine, Hangzhou, Zhejiang, China (People’s Republic); Zhejiang University School of Medicine, Hangzhou, Zhejiang, China (People’s Republic); The First Affiliated Hospital, Zhejiang University School of Medicine, Hangzhou, Zhejiang, China (People’s Republic); The First Affiliated Hospital, Zhejiang University School of Medicine, Hangzhou, Zhejiang, China (People’s Republic); The First Affiliated Hospital, Zhejiang University School of Medicine, Hangzhou, Zhejiang, China (People’s Republic)

## Abstract

Skeletal muscle aging is characterized by a progressive decline in muscle mass and function, yet its cellular and molecular mechanisms remain incompletely understood. In this study, we utilized single-nucleus RNA sequencing (snRNA-seq) to construct a comprehensive molecular atlas of skeletal muscle aging by analyzing vastus lateralis muscle samples from five Chinese male adults (aged 22 to 60 years) and three older adults (aged 99 to 101 years). Our analysis revealed significant muscle fiber atrophy in the older group, particularly a reduction in type IIA and IIX fibers, accompanied by a relative increase in RUNX1^+^, NCAM1^^+^^, and SORBS2^+^ fiber subpopulations. Differential gene expression and pathway enrichment analyses identified age-related alterations in metabolic pathways, calcium signaling, HIF-1, and MAPK pathways. Cell–cell communication analysis demonstrated an enhanced interaction network in aged muscle, with increased crosstalk between mast cells and fibro-adipogenic progenitors (FAPs), suggesting a role in extracellular matrix remodeling and chronic inflammation. Notably, ADGRL signaling was uniquely activated in type I and type II fibers in the older group, while CD99, NRG, and COLLAGEN signaling pathways were specifically upregulated, highlighting their potential roles in extracellular matrix remodeling and inflammation during aging. This study provides novel insights into the cellular diversity and molecular mechanisms underlying human skeletal muscle aging, offering potential therapeutic targets to mitigate age-related muscle decline.

